# Integrating Genome-Wide Association Study, Transcriptome and Metabolome Reveal Novel QTL and Candidate Genes That Control Protein Content in Soybean

**DOI:** 10.3390/plants13081128

**Published:** 2024-04-17

**Authors:** Xunchao Zhao, Hanhan Zhu, Fang Liu, Jie Wang, Changjun Zhou, Ming Yuan, Xue Zhao, Yongguang Li, Weili Teng, Yingpeng Han, Yuhang Zhan

**Affiliations:** 1 Key Laboratory of Soybean Biology in Chinese Ministry of Education (Key Laboratory of Soybean Biology and Breeding/Genetics of Chinese Agriculture Ministry), Northeast Agricultural University, Harbin 150030, China; zhaoxunchao2017@163.com (X.Z.); 17864701295@163.com (H.Z.); 18846913802@163.com (F.L.); w15536351237@163.com (J.W.); xuezhao@neau.edu.cn (X.Z.); yongguangli@neau.edu.cn (Y.L.); twlneau@163.com (W.T.); 2 Daqing Branch, Heilongjiang Academy of Agricultural Science, Daqing 163711, China; andazhouchangjun@163.com; 3 Qiqihar Branch, Heilongjiang Academy of Agricultural Science, Qiqihar 161006, China; yuanming0452@163.com

**Keywords:** soybean, genome-wide association study, transcriptome, metabolome, protein content

## Abstract

Protein content (PC) is crucial to the nutritional quality of soybean [*Glycine max* (L.) Merrill]. In this study, a total of 266 accessions were used to perform a genome-wide association study (GWAS) in three tested environments. A total of 23,131 high-quality SNP markers (MAF ≥ 0.02, missing data ≤ 10%) were identified. A total of 40 association signals were significantly associated with PC. Among them, five novel quantitative trait nucleotides (QTNs) were discovered, and another 32 QTNs were found to be overlapping with the genomic regions of known quantitative trait loci (QTL) related to soybean PC. Combined with GWAS, metabolome and transcriptome sequencing, 59 differentially expressed genes (DEGs) that might control the change in protein content were identified. Meantime, four commonly upregulated differentially abundant metabolites (DAMs) and 29 commonly downregulated DAMs were found. Remarkably, the soybean gene *Glyma.08G136900*, which is homologous with Arabidopsis hydroxyproline-rich glycoproteins (HRGPs), may play an important role in improving the PC. Additionally, *Glyma.08G136900* was divided into two main haplotype in the tested accessions. The PC of haplotype 1 was significantly lower than that of haplotype 2. The results of this study provided insights into the genetic mechanisms regulating protein content in soybean.

## 1. Introduction

Soybean is a significant cash crop with diverse applications, and its PC plays a pivotal role in determining the nutritional quality of food crops [1]. The percentage of soybean seeds in the PC amounted to approximately 40% [2]. The synthesis of soybean protein is a highly intricate process, involving multiple amino acids, and it has been observed that these amino acids play a crucial role in the development of crop seeds [3]. Soybean PC can be classified into three distinct groups based on their biological functions: storage proteins, structural proteins, and defense-related proteins, Notably, the storage proteins hold significant importance as edible proteins sources [4,5].

The regulation of PC involves the influence of multiple genes [6]. In order to uncover the genetic basis of soybean protein, many studies have been conducted. To date, a total of 248 QTLs associated with PC have been identified in the soybean genome database (https://www.soybase.org/) (accessed on 8 November 2023) [7]. Approximately 150 candidate genes have been identified as regulators of soybean seed PC [8]. The PC in soybean was assessed by researchers using a GWAS that analyzed over 2,597,425 single nucleotide polymorphisms (SNPs) across a diverse set of 264 (52 landraces and 212 improved varieties) accessions; a total of 11 QTLs were significantly associated with PC, and among them the QTL qPC-14 was detected in different environments [9]. Previous studies have conducted a GWAS analysis of the PC of 320 soybean materials, resulting in the identification of 15 novel QTL loci [10]. Recombinant inbred line populations were employed to conduct QTL analysis on PC: a total of four QTLS were identified, and qPSD20-1 and qPSD20-2 were found to exist stably in the two populations [11]. The cqProt-001 and cpProt-003 were identified for the first time on chromosomes 15 and 20 through quantitative trait locus (QTL) mapping of PC [12]. The quantitative trait locus (QTL) of soybean seed protein was identified from high protein line PI407788A, and the important protein QTL was found on chromosome 15 through fine mapping [13]. The CqSeed protein-003 was found to be significantly correlated with PC on chromosome 20 [14]. Previous researchers conducted QTL mapping analysis on the PC of 178 RILs populations, and a total of 50 QTLs were identified. Among them, qSPC_20-1 and qSPC_20-2 were repeatedly detected in multiple environments [6].

With the continuous development of multi-omics, joint analysis of gene expression and metabolites has been conducted. Research has found that soybean was primarily composed of eighteen amino acids, encompassing all nine essential amino acids [3]. It was observed that the expression levels of 7S and 11S were significantly upregulated during the late stages of soybean seed development [15,16]. The transcriptome and metabolome of soybean seeds with PC were comprehensively analyzed, resulting in the identification of 41,036 genes and 392 metabolites. Among them, a total of 12,712 DEGs and 315 DAMs were identified [17]. Previous studies have demonstrated that overexpression of *GmCGS2* leads to the upregulation of genes associated with the aspartic acid pathway, resulting in a substantial enhancement of total amino acid content and soluble protein levels within transgenic mature seeds [18]. In soybean, GmST05 may bind to GmSWEET10a in order to regulate seed size and protein content [19]. In rice, studies have found that GW2, GW5, GL3, and GS5 are closely related to grain weight and size [20]. Silencing the *OsGASR9* gene showed reduced grain size and yield [21]. In rapeseed, a total of 222 differential genes were identified to be related to seed weight [22]. In maize, the overexpression of *AtMAP18* resulted in elevated levels of zein and non-zein soluble proteins in the transgenic endosperm. Furthermore, an increased abundance of protein bodies was observed in the endosperm of transgenic maize compared to the wild-type [23]. Previous studies have demonstrated that the utilization of knockout mutants of TaGW2-B1 or combinations involving knockout mutations of TaGW2-A1 can effectively augment both thousand-grain weight (TGW) and grain protein content (GPC) in common wheat [24]. There are still few studies on the discovery of genes that regulate PC by combining genomics, transcriptomics, and metabolomics.

To elucidate the mechanism underlying soybean protein synthesis and enhance the PC of soybean seeds, an in-depth investigation was warranted. Data of genome, transcriptome and metabolome were collected based on an association panel of different soybean accessions. The PC of soybean was analyzed through GWAS using 266 soybean accessions. Then, we selected 30 accessions with different PC for transcriptome and metabolome analysis. Results of the GWAS, transcriptome and metabolome analyses were combined to determine the genetic determinants and related metabolites that regulate PC.

## 2. Results

### 2.1. Statistical and Variation Analysis of Protein Content

**Figure 1:**
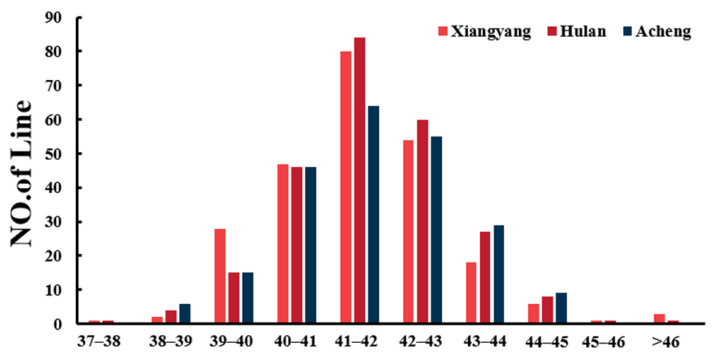
Frequency distribution of protein content (PC) in the three environments.

A total of 266 soybean accessions were examined, and the measurement of soybean PC was conducted at three locations (Xiangyang, Hulan, and Acheng) in 2021. The phenotypic variations of PC in the Xiangyang, Hulan, and Acheng environments ranged from 37.7% to 46.6%, 37.8% to 46.1% and 38.2% to 44.9% (Figure 1). The coefficient of variation (CV%) in three environments ranged from 3.1% to 3.2%, and the heritability of PC was 0.92 (Table S1). The results indicated that the PC was influenced by both genetic and environmental factors and was suitable for GWAS analysis.

### 2.2. Population Structure and GWAS Analysis

In this study, specific-locus amplified fragment sequencing (SLAF-seq) was used to sequence the genome of 268 soybean germplasms. A total of 23,131 high-quality markers (MAF ≥ 0.02, missing data ≤ 10%) were identified. SNPs were distributed on all 20 chromosomes in soybean (Figure S1A). By analyzing the principal components and kinship analyses of all SNPs, the evaluation of the variation of the first ten PC analyses showed an inflection point at PC3 (Figure S1B). This result indicated that the first three PCs dominated the population structure on the association mapping (Figure S1C). According to the pairwise relative-kinship coefficients of the association panel analysis, the 266 soybean accessions had a low genetic correlation within the population (Figure S1D).

### 2.3. Quantitative Trait Nucleotide (QTN) Associated with Protein Content by GWAS

**Figure 2:**
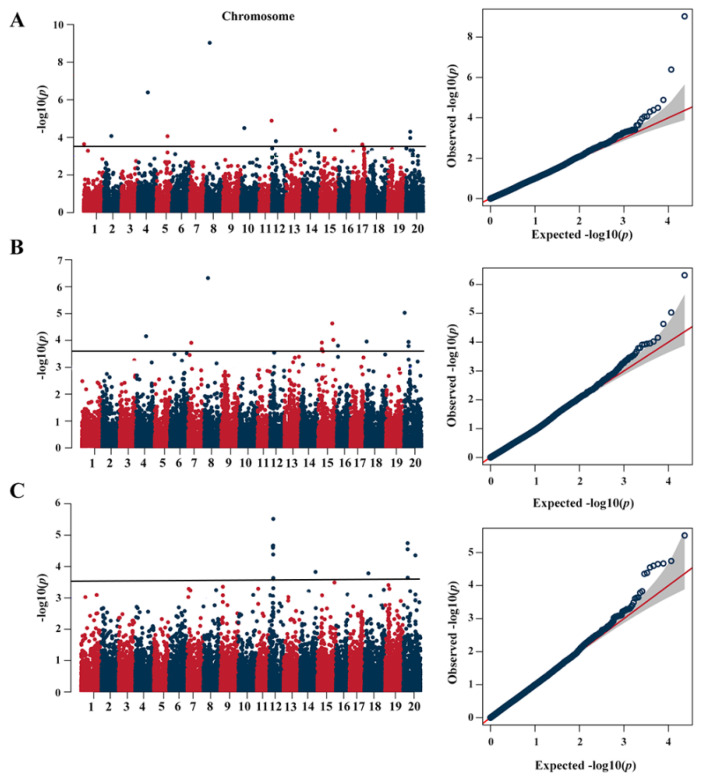
Manhattan and QQ plot of association mapping of PC in soybean. (**A**) Xiangyang in 2021. (**B**) Hulan in 2021. (**C**) Acheng in 2021. The dark-blue line on each subgraph indicates the log10 (*p* value) significance threshold.

In this study, a total of 31 signals were associated with PC, and were mainly distributed on chromosomes 1, 2, 4, 5, 6, 7, 8, 10, 11, 12, 14, 15, 16, 17, 18 and 20. Meantime, three SNP loci were identified in three or more environments, including rs12338 on Chr.12, rs22328 on Chr.20 and rs22326 on Chr.20. The remaining 28 QTNs were detected exclusively in one or two environments (Figure 2, Table 1). Among 31 QTNs, 25 QTNs were identified as located near the genomic regions of known QTLs related to soybean PC (Table 1). These results indicated that these QTNs were stable in different independent studies and should be reliable.

### 2.4. Transcriptomic Analysis of HPC and LPC Soybean Seeds

To elucidate the transcriptional regulation and identify potential candidate genes associated with PC, transcriptomic data of soybean seeds exhibiting high protein content (HPC) and low protein content (LPC) were generated using RNA-seq. A total of three comparison groups were established, including 5 HPC and 5 LPC varieties (FHPC vs. FLPC comparison group); 10 HPC and 10 LPC varieties (THPC vs. TLPC comparison group); and 15 HPC and 15 LPC varieties (HPC vs. LPC comparison group) (Table S2).

**Figure 3:**
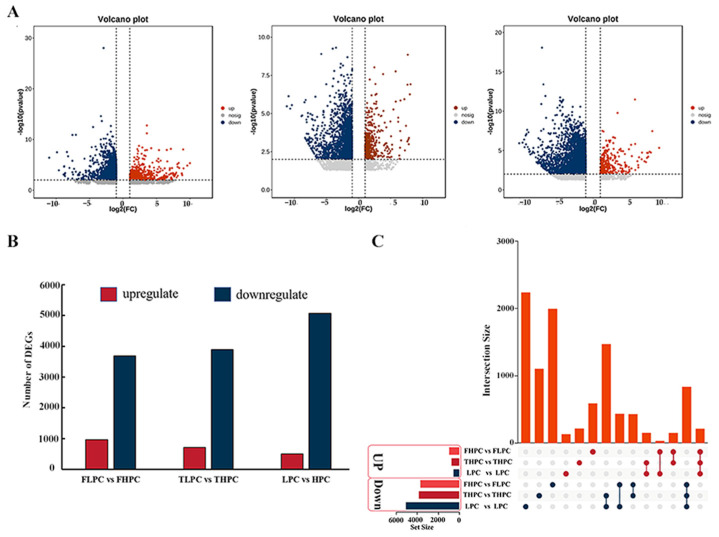
Multivariate statistical analysis of the transcriptome data in the soybean samples. (**A**) Volcano plot of differentially expressed genes (DEGs). (**B**) Number of DEGs in different comparison groups. Red and dark-blue columns represent the numbers of genes with upregulated and downregulated expression, respectively. (**C**) UpSetPlot diagram showing the overlapping DEGs in the three comparison groups.

A total of 4650, 4605, and 5580 DEGs were identified in FHPC vs. FLPC, THPC vs. TLPC, and HPC vs. LPC, respectively (Figure 3A,B; Table S3). In different comparison groups, the number of downregulated DEGs exceed that of upregulated DEGs (Figure 3B). Meantime, 209 commonly upregulated DEGs and 832 commonly downregulated DEGs were found across all three comparison groups (Figure 3C). The results indicated that variations exist in the levels of gene expression among diverse soybean varieties.

### 2.5. Combining GWAS and RNA Seq Results to Determine Candidate Genes for PC

To identify candidate genes regulating soybean PC, we integrated the findings from GWAS and RNA seq to select DEGs located within the 100 kbp region flanking the significant SNP loci (Table S4). In this study, five DEGs were identified on chromosomes eight and 12 (Table 2). According to functional annotation of the five candidate genes, *Glyma.08G136900* was directly homologous with *Arabidopsis AT2G27080* and considered a candidate gene involved in seed development [36]. *Glyma.08G136900* was designated as the *GmHRGP* gene, and has been reported to be involved in embryonic development [37,38]. Meanwhile, the expression level of *GmHRGP* gene was significantly upregulated in all three comparison groups. Furthermore, other *Arabidopsis* homologues were also defined as candidate genes based on gene annotation and expression levels, including basic helix–loop–helix (*Glyma.12G114300*), Haloacid dehalogenase-like hydrolase (*Glyma.08G137500*), and Nucleotide-sugar transporter (*Glyma.08G135800*) (Table 2, Figure S2).

In addition, 54 DEGs at other SNP loci were also detected, which potentially play a role in affecting soybean PC, including C3HC4-type RING finger protein (*Glyma.15G122900*), Transcription factor GTE6 (*Glyma.15G154600*), non-intrinsic ABC protein 9 (*Glyma.20G094400*), mitogen-activated protein kinase phosphatase 1 (*Glyma.11G243900*) and unknown protein (*Glyma.20G016300*) (Table S5).

### 2.6. Metabolic Profiling Analysis of MHPC and MLPC Soybean Seeds

**Figure 4:**
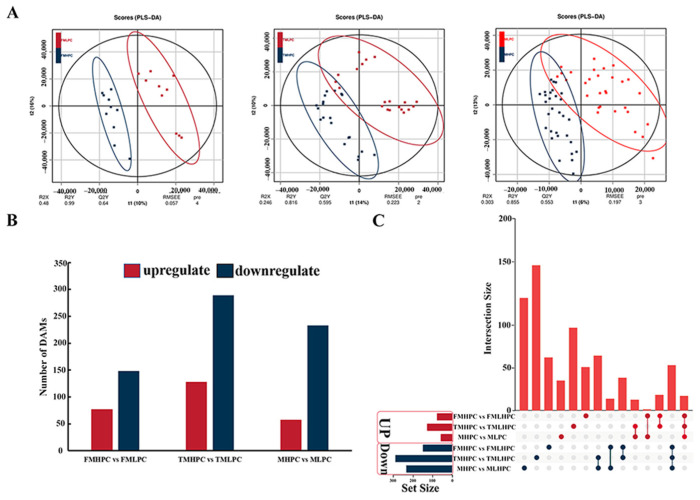
Multivariate statistical analysis of the metabolome data in the soybean samples. (**A**) OPLS-DA model analysis. (**B**) Number of DAMs in different comparison groups. Red and dark-blue columns represent the numbers of metabolites with upregulated and downregulated expression, respectively. (**C**) UpSetPlot diagram showing the overlapping DAMs in the three comparison groups.

To elucidate the changes in metabolites during the seed development stage under different protein levels, a non-targeted metabolic profiling analysis was conducted. A total of 15 high-protein-content (HPC) and 15 low-protein-content (LPC) soybean varieties were applied to compare changes in metabolites. The above samples were collected during the R6 developmental stage, and two biological replicates were obtained. Three comparison groups were established, including 5 high-protein-content and 5 low-protein-content varieties (MFHPC vs. MFLPC); 10 high-protein-content and 10 low-protein-content varieties (MTHPC vs. MTLPC), and 15 high-protein-content and 15 low-protein-content varieties (MHPC vs. MLPC) (Table S2). According to the analysis of the orthogonal partial least squares discriminant analysis (OPLS-DA) model, it was found that the model was stable and reliable (Figure 4A). A total of 70 DAMs were upregulated, and 291 DAMs were downregulated in MFHPC vs. MFLPC (Figure 4B). These metabolites were involved in lipids, secondary metabolites and unknown metabolites. In addition, a total of 202 and 322 DAMs were identified in MTHPC vs. MTLPC and MHPC vs. MLPC, respectively (Figure 4B). As shown in Figure 4C, a total of four commonly upregulated DAMs and 29 commonly downregulated DAMs were identified (Figure 4C).

### 2.7. Differential Accumulation of Metabolite with HPC and LPC 

In this study, the metabolic changes in high and low PC content in 30 soybean varieties during the R6 period were investigated. In FMHPC vs. FMLPC, a total of 25 DAMs were annotated into the KEGG pathway. Flavonoid and glycerophospholipid metabolism were annotated with the most DAMs (Table S5). In TMHPC vs. TMLPC, a total of 63 DAMs were annotated into the KEGG pathway. The flavonoid biosynthesis and purine metabolism annotated contained the most DAMs. In MHPC vs. MLPC, a total of 40 DAMs were annotated into the KEGG pathway and the purine metabolism and porphyrin and chlorophyll metabolism embraced the most DAMs (Table S6).

### 2.8. Co-Expression Analysis of Candidate Genes and DAM Metabolites

**Figure 5:**
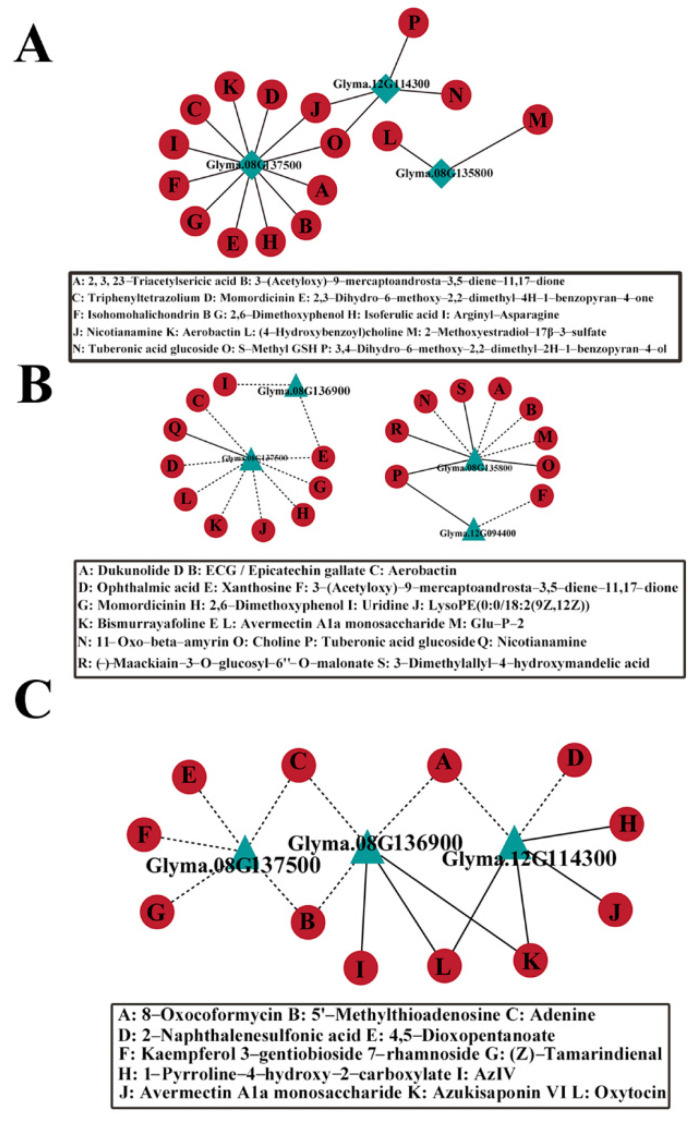
Network analysis of candidate genes and DAMs in the three comparison groups. (**A**) FHPC vs. FLPC, (**B**) THPC vs. TLPC, and (**C**) HPC vs. LPC. Red circles represent DAMs. Light green squares and triangles represent candidate genes. The solid line represents a positive correlation. The dashed line represents a negative correlation.

To explore the relationship between candidate genes and metabolites, a co-expression network analysis was constructed. In FHPC vs. FLPC, a total of 18 sub-networks were identified (|r| > 0.5, *p* < 0.05). *Glyma.08G042800* and *Glyma.12G094400* were positively associated with Nicotianamine (r > 0.93, *p* < 1.90 × 10^−9^ and r > 0.5, *p* < 0.02). It was speculated that the metabolite was closely associated with PC (Figure 5A). In THPC vs. TLPC, a total of 21 sub-networks were identified (|r| > 0.35). *Glyma.08G182500* was positively associated with 3-Dimethylallyl-4-hydroxymandelic acid (r > 0.65, *p* < 3.97 × 10^−6^). Meanwhile, it was found that 3-Dimethylallyl-4-hydroxymandelic acid is involved in the biosynthesis of Novobiocin. *Glyma.08G042800* was significantly associated with LysoPE(0:0/18:2(9Z,12Z)) (r < −0.35, *p* < 0.02). LysoPE(0:0/18:2(9Z,12Z)) belonged to glycerophospholipid metabolism (Figure 5B). In HPC vs. LPC, a total of 17 sub-networks were identified (|r| > 0.35). *Glyma.12G094400* was positively associated with oxytocin and Azukisaponin VI (r > 0.67, *p* < 4.02 × 10^−9^ and r > 0.53, *p* < 9.30 × 10^−6^). *Glyma.08G070300* was positively associated with oxytocin and Azukisaponin VI (r > 0.50, *p* < 3.22 × 10^−5^ and r > 0.45, *p* < 0.0002) (Figure 5C).

### 2.9. Gene-Based Association and Haplotype Analysis of Candidate Genes

**Figure 6:**
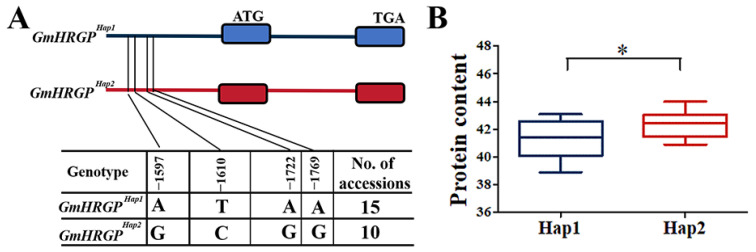
Haplotype analysis of genes with variations related to PC. (**A**) Schematic representation of the two haplotypes of *GmHRGP*. (**B**) Comparison of PC between two different haplotypes in 25 soybean accessions. * indicates significance at *p* < 0.05.

In order to further determine the relationship between candidate genes and traits, the SNPs of candidate genes were used for gene-based association analysis and haplotype investigation. Based on the association analysis, four SNPs were found in the promoter region of *Glyma.08G136900* (Figure 6A). SNP markers rs10468198, rs10468211, rs10468323 and rs10468370 were significantly associated with PC. *Glyma.08G136900* was mainly divided into two haplotype in the studied accessions. The PC of haplotype 1 was significantly lower than that of haplotype 2 (Figure 6B).

## 3. Discussion

The PC of soybean is an indicator that determines crop yield. Previous studies found that many QTN loci for PC were identified, and in comparison with our results, novel significant SNP loci have been detected (Table 1). Here, in a natural population of 266 soybean accessions by reduced-representation genome sequencing, a total of 23,149 high-quality SNPs were identified for GWAS analysis. The results revealed that a total of 40 SNPs had a significant association with PC. As we know, this is the first time that genomes, metabolomics and transcriptome have been used to analyze gene loci that control PC. We found that three overlapping SNPs were located at each location, and they were located near the genomic regions of known QTLs associated with soybean PC. This result demonstrates the reliability of our GWAS analysis.

Previous studies have identified many PC QTLs, but most of the identified QTLs were based on genetic background. Therefore, the new QTL/gene of PC still need to be further identified. In the study, a total of 40 QTNs located on 16 chromosomes were identified, which were associated with PC in three environments in 2021. In these QTNs, there were 33 QTNs which overlapped with or were near the known QTL (Table 1). Three QTNs (rs22328, rs22326 and rs12338) were significantly associated with PC, and the association between these three QTLs and PC has been reported [25,28,31]. Similarly, 30 QTNs were reported in one or two environments [12,25,26,27,28,29,30,31,32,33,34,35]. Additionally, a total of seven novel QTNs associated with PC were identified.

To date, using RNA seq for screening candidate genes in GWAS has emerged as the predominant approach. In recent years, the integration of GWAS and RNA-seq has been employed to identify novel genes associated with complex agronomic traits. For instance, the identification of four candidate genes potentially involved in the regulation of root development in rapeseed was achieved through a combination of GWAS and RNA-seq analyses [39]. This study identified four potential candidate genes (*Glyma.08G136900*, *Glyma.12G114300*, *Glyma.08G135800* and *Glyma.08G137500*) which were associated with PC using this methodology. The above genes exhibited upregulated expression in all three comparison groups. In this study, it was found that *Glyma.08G042800* was significantly associated with LysoPE(0:0/18:2(9Z,12Z)) (r > −0.35, *p* < 0.02). Meantime, LysoPE(0:0/18:2(9Z,12Z)) belonged to glycerophospholipid metabolism. It was reported that a negative correlation existed between the soybean oil content and the PC [40]. We also found that *Glyma.08G042800* and *Glyma.12G094400* were positively associated with Nicotianamine (r > 0.93, *p* < 1.90 × 10^−9^ and r > 0.5, *p* < 0.02). It has been reported that the SlALMT4 and SlALMT5 play crucial roles in the seed development process of fruits [41]. Therefore, the above research could provide valuable genetic insights into the synthesis mechanism of soybean PC.

The *Glyma.08G136900* encoded the hydroxyproline-richglycol protein. It was homologous with *Arabidopsis AT2G27080*, which belongs to the HRGPs family. The HRGP family is a very interesting gene family, with its members exhibiting diverse fundamental functions [42]. According to previous reports, the *GmHRGP* gene has been found to be involved in cell development [43]. In this study, we identified two haplotypes in the transcription-regulating region of the *GmHRGP* gene, namely *GmHRGP ^Hap1^* and *GmHRGP ^Hap2^*. More and more evidence shows that the natural variation in the promoter region exerts a significant influence on the expression levels of regulatory genes. The variation in the *GmST05* promoter region sequence leads to an increase in soybean grain size [19]. The PC of *GmHRGP ^Hap1^* was significantly lower than that of *GmHRGP ^Hap2^*, indicating a pivotal role played by *GmHRGP ^Hap2^* in enhancing the PC. Therefore, a modification of the transcription-regulating regions of *GmHRGP* may help us improve soybean PC.

## 4. Materials and Methods

### 4.1. Plant Materials

The natural population of 266 soybean germplasms were used in this study. Soybean seeds were planted in a random grouping manner. All materials were planted in three locations in Harbin, including Acheng, Xiangyang and Hulan districts (2021) (Table S7). Each plot contained single-row plots, 2 m long with 34 plants per row, and with a threefold repetition per location. Mature soybean plants were harvested and the PC in mature seeds was determined using Infratec 1241 NIR Grain Analyzer (FOSS, Hoganas, Sweden).

Thirty soybean varieties including 15 high-PC (HPC: 43.5–48.2%) and 15 low-PC (LPC: 37.7–39.4%) varieties were obtained from the Soybean Research Institute of Northeast Agricultural University. Thirty soybean germplasms were planted under the same field design as that of the natural population in Harbin, Heilongjiang, China (Table S2). Transcriptome and metabolome samples were collected during the R6 developmental stage, with two biological replicates obtained. Subsequently, all samples were rapidly frozen in liquid nitrogen.

### 4.2. DNA Isolation and SNP Genotyping Data Collection

Previous methods were used for DNA isolation and SNP genotyping analysis [44]. Briefly, the genomic DNA of the test samples was extracted using the hexadecyl trimethyl ammonium bromide (CTAB) method, using the reduced-sequencing method for genotyped genomic DNA. The restriction digestion enzymes MseI and HaeIII (Thermo Fisher Scientific Inc., Waltham, MA, USA) were selected to generate a minimum of 50,000 sequencing tags per tested sample, with lengths ranging from approximately 300 bp to 500 bp. The sequencing libraries in each accession were constructed based on the obtained sequencing tags, which covered unique genomic regions in soybean. The barcode method and Illumina Genome Analyzer II System (Illumina Inc., San Diego, CA, USA) were employed to generate 45-bp sequence reads at both ends of the sequencing tags from each accession library. The alignment of the obtained raw paired-end reads with the reference genome (Glycine max Wm82. a2. v1) was performed using BWA software (Version: 0.6.1-r104). SAMtools 48 software was employed to convert the mapping results into the BAM format and to effectively filter out unmapped and non-unique reads. The BEDtools software (Version: 2.17.0) was utilized to calculate the sequence alignment coverage. The detection of SNPs was performed using the GATK (version 2.4-7-g5e89f01) and SAMtools software. The SNP annotation was conducted based on the soybean genome (Glycine max Wm82. a2. v1) using the ANNOVER package. A total of 23,131 high-quality SNPs (minor allele frequency ≥ 0.02, missing rate ≤ 10%) were used for analysis.

### 4.3. Genome-Wide Association Analysis

The association signals of PC were identified using the compressed mixed linear model (CMLM) with default parameters in the GAPIT package, based on a total of 23,131 SNPs obtained from 266 tested samples [45]. The *p* value was calculated using the Bonferroni method, with a significance level of α ≤ 0.05 (−log10(*p*) > 3.5), and served as the threshold for determining the presence of a significant association signal [44]. Following the GWAS result, the visualization of the Manhattan plots was generated through R package ‘qq man’ [46].

### 4.4. Metabolome Analysis

Non-targeted metabolome analysis was accomplished by Bioacme Biotechnology Co., Ltd. (Wuhan, China). A total of 100 mg of samples of soybean seeds at R6 stage were placed in a 1.5 mL centrifuge tube, and 300 μL of 75% methanol/water mixed solvent (0.1% formic acid, *v*/*v*) was added and swirled for 30 s. After 15 min of ultrasound in a water bath at 20 °C, the samples were swirled for 2 min. After centrifugation at 12,000 rpm for 20 min at 4 °C, supernatant was taken and tested by high-performance liquid chromatography combined with quadrupole time-of-flight mass spectrometry (HPLC-Q-TOF/MS, 6545 Q-TOF LC/MS, Agilent, Santa Clara, CA, USA). Chromatographic conditions: mobile phase A was water (containing 0.1% formic acid), mobile phase B was acetonitrile (containing 0.1% formic acid), flow rate was 0.20 mL·min^−1^, injection volume was 1 μL, and column temperature was 30 °C. Collection mode: ESI (+/−), capillary voltage of 4.0 kV (positive ions), 3.5 kV (negative ions), gas temperature of 300 °C, drying gas flow rate of 9 L/min, nebulizer pressure of 35 psi, sheath gas temperature of 350 °C, sheath gas flow rate of 11 L/min, and mass range of *m*/*z* 100–1700. The original mass spectrometry data were analyzed using Agilent Profiler software 2023 (Agilent Technologies, Santa Clara, CA, USA). The differential metabolites were analyzed using the OPLS-DA (orthogonal partial least squares–discriminant analysis) model, with a VIP score of ≥1 and a |log2 (fold change)| of ≥1, utilizing the ropls packages. The metabolome analysis was conducted using a total of 60 samples, which were derived from two biological replicates.

### 4.5. Transcriptome Analysis

At the R6 stage of soybean development, total seed RNA was extracted using TRIzol reagent (Invitrogen, Carlsbad, CA, USA) and purified to isolate the mRNA. After the RNA samples were qualified, eukaryotic mRNA was enriched using magnetic beads coated with Oligo(dT) (Thermo Scientific, USA). The cDNA library was constructed using the Illumina HiSeq platform by adding a fragmentation buffer to break the mRNA into short fragments, synthesizing the double-stranded cDNA using mRNA as a template, and then purifying the double-stranded cDNA using AMPure XP beads (Beckman Coulter, Brea, CA, USA). Further PCR amplification was performed, and the PCR product was purified using AMPure XP beads to obtain a strand-specific cDNA library. High-quality readings were mapped to reference genomes (Glycine max Wm82.a2.v1) by Hisat2 software (version 2.2.1). Differentially expressed genes were screened using R package DESeq2 with |log2Fold change| > 1 and *p* < 0.05 was used as the standard [47,48,49]. The transcriptome analysis was conducted using a total of 60 samples, which were derived from two biological replicates.

### 4.6. Prediction of Candidate Genes

The upstream and downstream 100 kb genomic regions of each significant SNP in the GWAS results were defined as putative candidate genes [44]. To determine candidate genes controlling the PC of soybean seeds, a comprehensive approach integrating GWAS and RNA-seq analysis was performed to identify DEGs within confidence intervals and annotate their functions using Arabidopsis homologues. A total of 10 high-PC and 15 low-PC lines were screened from genome re-sequencing data to identify candidate gene variations, including exons, 5′UTRs, 3′UTRs and promoter regions. The identification of haplotypes was performed through gene-based association analysis using the General Linear Model (GLM) method in TASSEL software (version 3.0) [50].

### 4.7. Co-Expression Analysis

The correlation coefficient was calculated between candidate genes and metabolites using Perl script, and Pearson’s correlation cut-off value of |r| > 0.35, *p* < 0.05 was established. The visualization of co-expression networks was completed using the Cytoscape package (version 3.8.2) [51].

### 4.8. Quantitative Real-Time PCR

Several differential genes were screened and analyzed by Quantitative Real-Time PCR. Total seed RNA was extracted using TRIzol reagent (Invitrogen), and cDNA was generated by ReverTra Ace qPCR RT Master Mix (TOYOBO, Osaka, Japan). Real-Time PCR was performed using a Bio-Rad CFX96 quantitative PCR instrument with a SYBR Select Master Mix RT-PCR system (TOYOBO, Osaka, Japan). Relative expression levels were estimated using the 2^−ΔΔct^ method [52]. *GmACTIN4* were used as internal control. All qRT-PCR primers obtained are shown in Table S8.

## Figures and Tables

**Table 1:** Single nucleotide polymorphisms (SNPs) associated with PC of soybean and known QTL overlapped with peak SNP.

Locus Name	Environmental ^a^	Chr ^b^	Position	Effect	−Log10 (*p*)	MAF	Known QTLs
rs8158	E1, E2	8	10,476,797	1.32	9.03/6.32	0.02	[25]
rs4186	E1, E2	4	27,955,520	2.02/1.49	6.39/4.16	0.02	[26]
rs12036	E1	11	33,830,623	−1.12	4.89	0.04	
rs10510	E1	10	9,242,053	−1.493	4.5	0.04	[27]
rs16341	E1, E2	15	41,855,327	1.1111111	4.38/4.02	0.04	
rs22328	E1, E2, E3	20	12,157,089	0.68/0.63/0.72	4.30/3.79/4.74	0.12	[28]
rs1724	E1	2	20,263,396	0.83	4.07	0.07	[29]
rs5276	E1	5	30,572,600	1.16	4.06	0.05	[30]
rs22326	E1, E2, E3	20	12,137,051	0.64/0.64/0.69	3.97/3.94/4.55	0.13	[31]
rs12338	E1, E2, E3	12	11,269,928	0.41/0.38/0.45	3.80/3.54/5.52	0.39	[25]
rs34	E1	1	858,919	−0.63	3.64	0.11	[31]
rs18647	E1	17	28,541,923	−0.54	3.62	0.37	[30]
rs22112	E2	20	1,396,122	0.94	5.03	0.07	[32]
rs16229	E2	15	38,871,682	1.09	4.63	0.04	[29]
rs19145	E2	18	3,775,534	−1.27	3.96	0.03	[33]
rs15253	E2	15	9,792,514	−0.44	3.92	0.38	
rs7157	E2	7	8,398,920	0.49	3.91	0.25	
rs16837	E2	16	3,244,871	−0.78	3.8	0.07	
rs15251	E2	15	9,752,108	−0.42	3.68	0.42	
rs15349	E2	15	12,923,409	−0.85	3.6	0.06	
rs6741	E2	6	47,134,144	0.73	3.52	0.08	[34]
rs12331	E3	12	10,978,300	0.56	4.67	0.18	[25]
rs12316	E3	12	9,835,536	−0.43	4.65	0.43	[25]
rs12325	E3	12	10,505,242	−0.47	4.6	0.26	[25]
rs12329	E3	12	10,956,419	−0.51	4.38	0.19	[25]
rs22678	E3	20	33,768,863	0.44	4.36	0.38	[12]
rs14891	E3	14	4,366,8761	−0.8	3.83	0.05	[33]
rs19362	E3	18	11,173,355	−0.68	3.78	0.08	[30]
rs22335	E3	20	12,352,049	−0.62	3.64	0.1	[35]
rs12340	E3	12	11,328,123	0.45	3.63	0.25	[25]
rs12330	E3	12	10,960,320	0.47	3.61	0.17	[25]

^a^ E1: at Xiangyang in 2021; E2: at Hulan in 2021; E3: at Acheng in 2021. ^b^ Chr: Chromosome.

**Table 2:** Candidate DEGs that are consistent in three comparison groups.

Gene ID	Marker	Chr	Position (bp)	FHPC vs. FLPC (Log2FC)	THPC vs. TLPC (Log2FC)	HPC vs. LPC (Log2FC)	Arabidopsis Homologs	Gene Function
Glyma.08G137500	rs8158	8	10,476,797	3.17	1.37	1.93	AT5G65140	Haloacid dehalogenase-like hydrolase (HAD) superfamily protein
Glyma.08G136900	rs8158	8	10,476,797	4.66	2.83	4.27	AT2G27080	Late embryogenesis abundant (LEA) hydroxyproline-rich glycoprotein family
Glyma.08G135600	rs8158	8	10,476,797	−1.20	−1.12	−1.65	AT5G10250	Phototropic-responsive NPH3 family protein
Glyma.08G137900	rs8158	8	10,476,797	−1.32	−1.58	−1.09	AT4G39790	Protein of unknown function (DUF630 and DUF632)
Glyma.12G114100	rs12338	12	11,269,928	6.26	NA	NA	AT4G28350	Concanavalin A-like lectin protein kinase family protein
Glyma.08G135800	rs8158	8	104,76,797	2.89	1.97	2.46	AT5G65000	Nucleotide-sugar transporter family protein
Glyma.12G114300	rs12338	12	11,269,928	2.77	1.22	1.33	AT4G20970	basic helix–loop–helix (bHLH) DNA-binding superfamily protein

## Data Availability

Data are contained within the article and Supplementary Materials.

## Supplementary Materials

The following supporting information can be downloaded at: https://www.mdpi.com/article/10.3390/plants13081128/s1, Figure S1: Genetic features of the mapping population. (A) The density distribution of SNPs. (B) The first three principal components reflected by SNPs used in the GWAS. (C) Population structure of soybean germplasm. (D) A heatmap of the kinship matrix of the 266 soybean accessions. Figure S2: Analysis of candidate genes by qRT-PCR. Table S1: Statistical analysis of protein content in soybean. Table S2: Protein content of thirty soybean varieties. Table S3: The DEG gene functional annotation. Table S4: Genes in 100 kbp flanking regions of peak SNP associated with protein content of soybean. Table S5: The candidate genes for protein content by combining GWAS and RNA-seq. Table S6: Differential metabolites in three comparison groups. Table S7: The information of the soybean association panel. Table S8: Primers used for qRT-PCR.
