# Supplementary material for: Integrating Genome-Wide Association Study, Transcriptome and Metabolome Reveal Novel QTL and Candidate Genes That Control Protein Content in Soybean
Source: Plants (Basel). 2024 Apr 17;13(8):1128. doi: 10.3390/plants13081128 (PMC11054237; doi:10.3390/plants13081128)
Supplement: Supplementary file 1 [file plants-13-01128-s001.zip › Table S1.pdf]

Table S1. Statistical analysis of protein content in soybean.

Trait	Location	Min(%)	Max(%)	Mean(%)	SD$_a$	CV$_b$(%)	Heritability
Protein content	Xiangyang	37.7	46.4	41.5	1.35	3.20%	0.92
	Hulan	37.8	46.1	41.6	1.3	3.10%	
	Acheng	38.2	44.9	41.7	1.29	3.10%	

[a] SD: Standard Deviation; [b] CV: Coefficient of Variation;
