# Supplementary figures and images for: Integrating Genome-Wide Association Study, Transcriptome and Metabolome Reveal Novel QTL and Candidate Genes That Control Protein Content in Soybean
Source: Plants (Basel). 2024 Apr 17;13(8):1128. doi: 10.3390/plants13081128 (PMC11054237; doi:10.3390/plants13081128)

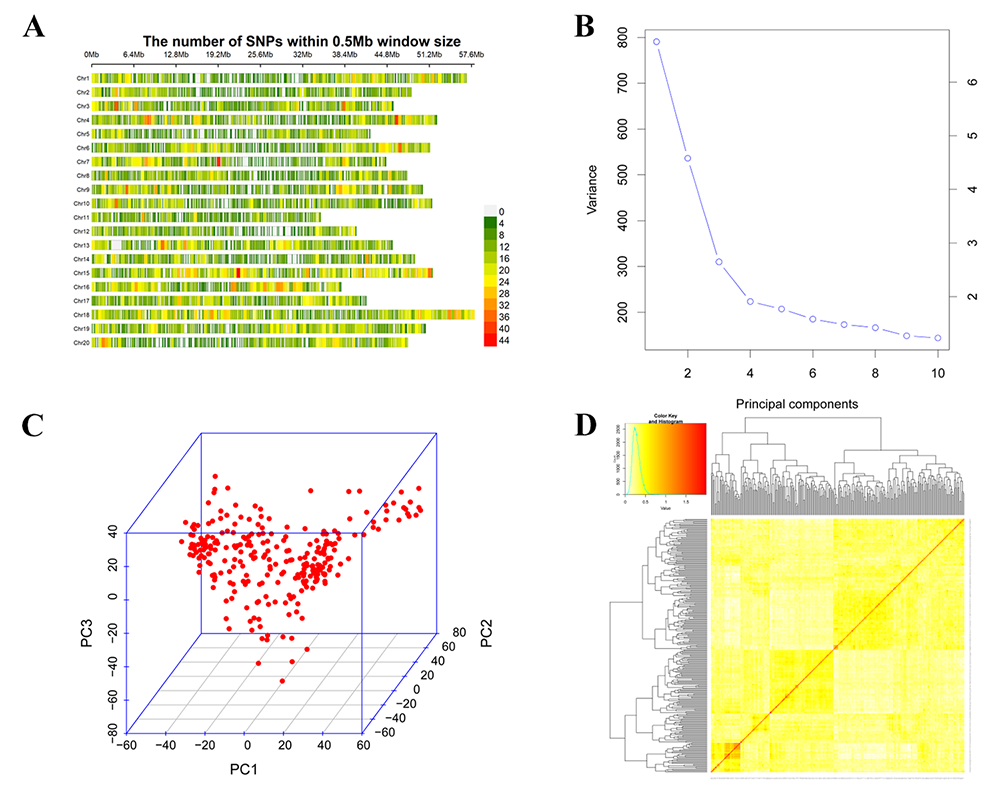

Supplement: Supplementary file 1 [file plants-13-01128-s001.zip › Figure S1.tif]

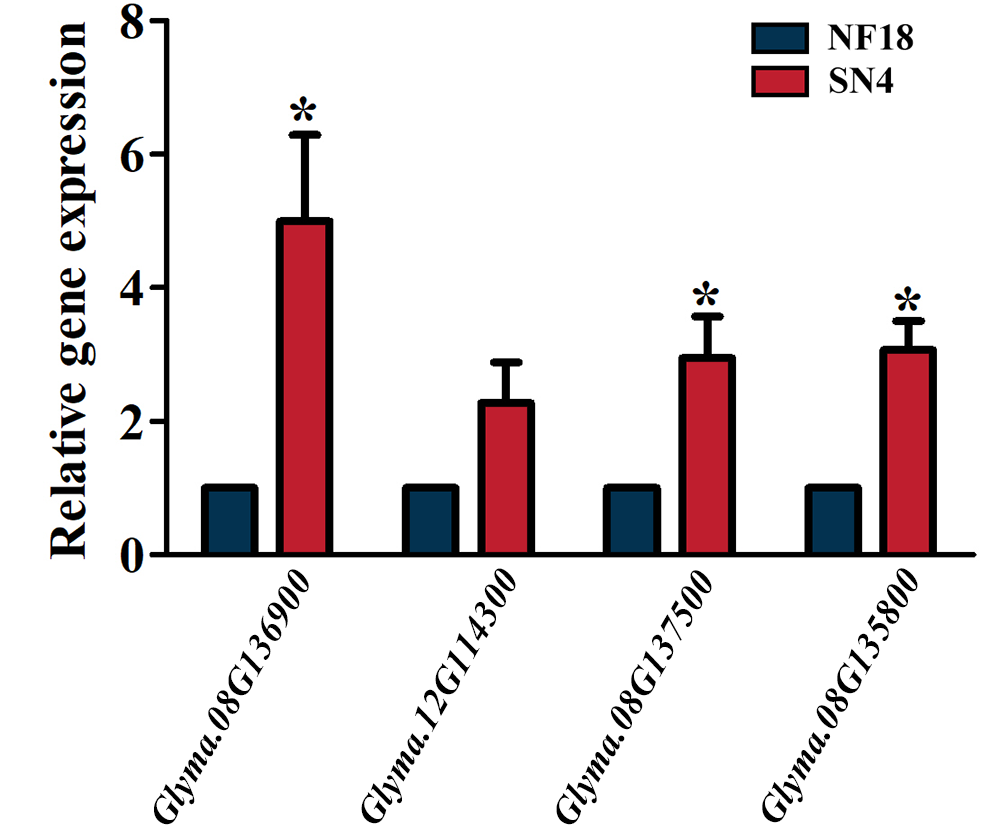

Supplement: Supplementary file 1 [file plants-13-01128-s001.zip › Figure S2.tif]
